# Reduction in Endometrioma Size with Three Months of Aromatase Inhibition and Progestin Add-Back

**DOI:** 10.1155/2015/878517

**Published:** 2015-07-12

**Authors:** Sanjay K. Agarwal, Warren G. Foster

**Affiliations:** ^1^Center for Endometriosis Research and Treatment, Department of Reproductive Medicine, UC San Diego, 9500 Gilman Drive, No. 0633, La Jolla, CA 92093-0633, USA; ^2^Department of Obstetrics and Gynecology, McMaster University, 1280 Main Street West, HSC-3N52D, Hamilton, ON, Canada L8S 4K1

## Abstract

The purpose of this study was to assess the impact of 3 months of aromatase inhibition together with progestin add-back on ovarian endometrioma size. This prospective cohort study was performed at University Medical Center (UC San Diego). Women trying to conceive were excluded. After informed consent, all women were treated with the aromatase inhibitor letrozole (5 mg/d) with norethindrone acetate (5 mg/d) add-back for 3 months. Pre- and posttreatment assessments of endometrioma sizes were performed by ultrasound. The impact of treatment on pain was determined using the patient assessed endpoints of the Biberoglu and Behrman scale. These included assessing dysmenorrhea, dyspareunia, and nonmenstrual pelvic pain each on a scale from 0 to 3. The primary endpoint of this study was the change in ultrasound documented endometrioma size. Fourteen endometriomas in 8 consecutive women were treated for 3 m. Mean endometrioma diameter decreased 50% from 4.6 ± 1.6 cm to 2.3 ± 1.6 cm (mean ± SD). This represents a 75% decrease in endometrioma volume. Endometriosis symptoms of dysmenorrhea, dyspareunia, and nonmenstrual pelvic pain also improved with treatment. In conclusion, a 3-month course of high dose aromatase inhibition with progestin add-back significantly reduces ovarian endometrioma size and warrants further investigation.

## 1. Introduction

Endometriosis is the presence of endometrial tissue outside the uterus. As is the case with normal endometrium, endometriosis is also estrogen sensitive. It undergoes proliferation in the presence of estrogen and atrophy in its absence. Although endometriosis lesions are typically small implants commonly found on pelvic structures such as the bladder, ovaries and in the cul-de-sac, they can sometimes form larger, cystic lesions, which are called endometriomas. By far the most common location for endometriomas is the ovaries. Although it is unclear why endometriomas predominantly develop at this site, it is assumed to be at least in part because the ovary is the major site of estradiol production.

Endometriomas are not believed to regress spontaneously. Therefore in order to prevent the possibility of ovarian torsion or cyst rupture, endometriomas are commonly managed by surgical excision, which is both expensive and invasive. Moreover, while there is minimal risk associated with surgery, the complications associated with surgery are potentially serious. In addition, excising endometrioma has been shown to decrease ovarian reserve as judged by elevation in FSH (Ferrero et al. 2012) and decreases in AMH [1, 2], antral follicle count [3], and number of oocytes retrieved for subsequent IVF [4]. Although surgical excision of endometriomas may have detrimental repercussions for those contemplating pregnancy, the presence of ovarian endometriomas at time of oocyte retrieval for in vitro fertilization poses risks of pelvic infection after oocyte retrieval and oocyte culture contamination by endometriotic fluid [5–7]. Consequently, women with endometriomas undergoing IVF and their healthcare providers are faced with a unique and troubling situation whereby excising endometrioma decreases ovarian reserve thus compromising both natural and assisted fertility yet leaving endometriomas in place prior to IVF threatens to increase the risk of pelvic infection and contaminate the culture system thereby compromising results of this expensive treatment.

Endometriosis contains the aromatase enzyme [8], inhibition of which shrinks endometriosis lesions in postmenopausal women [9]. The location of this enzyme appears to have preponderance for endometriomas. Indeed, a recent study revealed that aromatase expression and tissue estrogen concentrations are profoundly higher in endometriomas compared to peritoneal and deep infiltrating endometriotic lesions [10]. The first successful use of an aromatase inhibitor was documented in a postmenopausal woman with a recalcitrant endometriotic lesion [11]. In this case, inhibition of aromatase reduced circulating estradiol concentrations and induced regression of the endometriotic implant. Although no medical therapy has been conclusively shown to shrink endometriomas, one study of 5 women did demonstrate shrinkage of recurrent endometriomas with a 6 m course of aromatase inhibition [12]. Because of the reduction in circulating estradiol levels, use of an aromatase inhibitor has been associated with menopausal symptoms. The use of add-back is now a common strategy to prevent hypoestrogenic symptoms that would otherwise occur with estrogen suppression. That study used a 30 mcg oral contraceptive for this purpose.

The primary objective of this study was to determine if a three-month course of the aromatase inhibitor letrozole (5 mg daily) together with the add-back approved by the FDA for use with GnRHa, 5 mg norethindrone acetate daily, could reduce endometrioma size in premenopausal women. Secondary objectives included changes in patient reported endpoints of dysmenorrhea, dyspareunia, and nonmenstrual pelvic pain (NMPP) with treatment.

## 2. Materials and Methods

This study was approved by the UC San Diego IRB and performed at the UC San Diego Center for Endometriosis Research and Treatment, in the Department of Reproductive Medicine.

Eight consecutive women wishing to try the off-label use of letrozole were enrolled. In these women, 14 endometriomas were assessed. Inclusion criteria for being offered this unproven therapy included not wishing to conceive over the ensuing 6 months, ultrasound imaging consistent with endometriomas, at least one endometrioma >3.0 cm mean diameter as well as age >18 years and <45 years. Exclusion criteria were women desiring pregnancy, contraindication to either letrozole or norethindrone acetate, and those in whom there was doubt as to the presence of an endometrioma. All women in this study wished to preserve fertility. After informed consent to try an off-label and unproven therapy for the possible reduction in endometriomas size, each woman received a 3-month off-label course of daily 5 mg letrozole with 5 mg norethindrone acetate add-back. Because of the relatively short course of therapy a higher, 5 mg daily dose of letrozole was used rather than the more typical and lower 2.5 mg daily dose. And as is typically the case, use of add-back was intended to minimize the negative effects of induced hypoestrogenemia and thus make the letrozole therapy more tolerable. All women were asked to use barrier contraception. Pre- and posttreatment assessments of endometrioma sizes were performed by transvaginal ultrasonography using GE Voluson machines. The mean of the largest two perpendicular diameters of each endometrioma was recorded. Impact of this treatment on pain was determined using the three patient reported endpoints of the standard Biberoglu and Behrman scale [13]. These included assessing dysmenorrhea, dyspareunia, and nonmenstrual pelvic pain each on a scale from 0 to 3, thus providing an overall pelvic pain symptom score range from 0 to 9. Pain assessments were recorded prior to initiating treatment and after 3 months of therapy.

### 2.1. Statistical Methods

JMP statistical software and Microsoft excel were used for the analyses. Because of the nonnormal distribution of endometrioma sizes, the nonparametric two-tailed Wilcoxon signed-rank test was used to compare paired pre- and posttreatment endometrioma sizes. Unless otherwise noted, data are shown as mean ± SD. A *P* value ≤ 0.05 was considered significant.

## 3. Results and Discussion

Eight consecutive women with a total of 14 endometriomas were enrolled. Their demographics are shown in Table 1.

**Figure 1:**
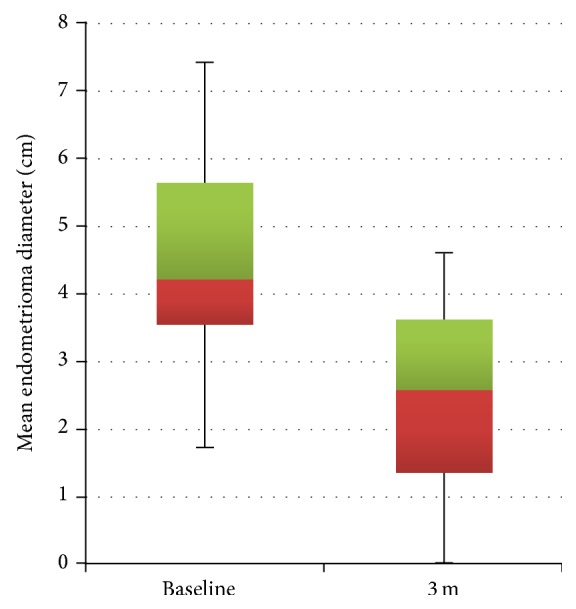
Distribution of endometrioma diameter quartiles prior to and after treatment with 3 months of aromatase inhibition plus progestin add-back.

**Figure 2:**
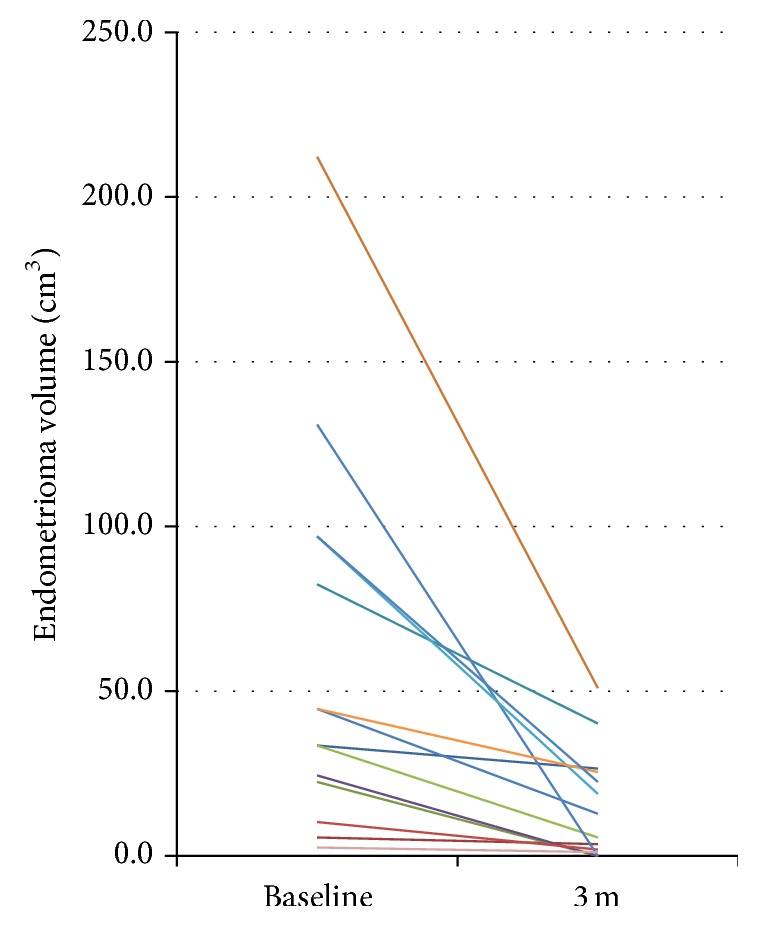
Change in individual endometrioma volumes. Mean endometrioma volume decreased 75% from 60.1 cm^3^ to 15.0 cm^3^ with 3 months of aromatase inhibition plus progestin add-back.

With three months of treatment, mean endometrioma diameter decreased 50% from 4.6 ± 1.6 cm, range from 1.7 to 7.4 cm, to 2.3 ± 1.6 cm, range 0–4.3 cm (*P* < 0.01) (Figure 1). The change in diameter corresponds to a mean endometrioma volume reduction of 75% from 60.1 ± 58.7 cm^3^, range 2.6–212.2 cm^3^, to 15.0 ± 16.4 cm^3^, range 0–51 cm^3^ (*P* < 0.01). Aromatase inhibition induced a reduction in individual implant volume (Figure 2).

With treatment, there was a reduction in patient reported symptom endpoints of the Biberoglu and Behrman scale with mean dyspareunia score decreasing from 2 to 0 and mean dyspareunia and nonmenstrual pelvic pain scores decreasing from 1 to 0.

Thus far there has been minimal data showing that premenopausal ovarian endometriomas can be managed with hormonal manipulation. The current pilot study shows for the first time that a 3 m course of aromatase inhibition with progestin add-back can produce a mean of 75% decrease in endometrioma volume. Results of this study highlight the feasibility of shrinking ovarian endometriomas with a relatively short, 3 m course of a daily administered aromatase inhibitor letrozole (5 mg) and the progestin norethindrone acetate (5 mg).

Aromatase appears to be a key enzyme in endometriosis. Within the lesions, it leads to the production of estrogen, which in turn stimulates cyclooxygenase-2, thus resulting in increased prostaglandin *E*_2_ formation. In turn, PGE_2_ stimulates aromatase expression and activity, thus producing a positive feedback loop [14, 15]. This dysregulation of aromatase appears to be particularly the case with regard to endometriomas [10].

Although endometriosis affects fertility through multiple mechanisms, it is possible that women with ovarian endometriomas represent a unique subgroup. For example, it has been proposed that the mere presence of endometriotic cysts on the ovary leads to increased ovarian inflammation and microscopic structural changes in the ovarian cortex such that there is a reduced follicular density [16]. The authors suggest consideration of surgical intervention even at an early stage of cyst formation for the protection of ovarian reserve. Based on our findings and the accumulating data indicating that surgery on endometriomas further reduces ovarian reserve, we propose that the impact of medical treatment for endometriotic cysts also be comprehensively evaluated with regard to ovarian reserve parameters.

The economic cost of managing endometriosis can be substantial. For example, the annual healthcare dollar expenditure on endometriosis [17, 18] is comparable to that spent on other chronic conditions such as Crohn's disease, rheumatoid arthritis, and asthma. This expense is likely related to both the chronic nature of the disease and surgical management that is often required. By reducing the need for surgical intervention, the overall cost of managing endometriosis should be reduced. In general terms, the replacement of a surgical treatment by one that is medical is a natural and desired evolution of healthcare as pathophysiology is better understood.

Although our findings are encouraging, larger double blinded, placebo controlled randomized trials will be required to verify these preliminary findings. Additional relevant questions also need to be addressed including optimal dose and duration of therapy as well as determination of any impact on ovarian reserve. It would also be helpful to determine the duration of effect. Further, as a strategy prior to IVF for the purposes of reducing the risks of pelvic infection after oocyte retrieval and gamete culture medium contamination, assessment of the impact of this strategy on IVF complications and on pregnancy outcomes would be useful.

## 4. Conclusions

In conclusion, we show here for the first time that a 3 m course of aromatase inhibition plus progestin significantly decreases ovarian endometrioma size. The major healthcare implications of this finding are (1) reducing costs and invasiveness in the management of these common ovarian cysts by possibly converting what was a surgical management to one that is medical and (2) the prospect of improved safety and better outcomes for women with endometriomas undergoing in vitro fertilization.

## Figures and Tables

**Table 1:** Baseline demographics of study population.

	Mean ± SD	Range
Age/years	32.4 ± 7.6	23–41
BMI/kg/m^2^	20.6 ± 2.3	16.7–23.2
Number of previous providers consulted for pain	2.3 ± 1.3	1–4
Time since onset of symptoms/years	8.2 ± 8.4	1–22
Mean baseline endometrioma diameter/cm	4.6 ± 1.6	1.7–7.4
Mean baseline endometrioma volume/cm^3^	60.1 ± 58.7	2.6–212.2
